# Passive repetitive stretching is associated with greater muscle mass and cross-sectional area in the sarcopenic muscle

**DOI:** 10.1038/s41598-021-94709-0

**Published:** 2021-07-27

**Authors:** Yumin Wang, Satoshi Ikeda, Katsunori Ikoma

**Affiliations:** 1 Department of Rehabilitation Medicine, Hokkaido University Graduate School of Medicine, Kita 15, Nishi 7, Kitaku, Sapporo-shi, Hokkaido 060-8638 Japan; 2 Department of Rehabilitation Medicine, Hokkaido University Hospital, Kita 14, Nishi 5, Kitaku, Sapporo-shi, Hokkaido 060-8648 Japan

**Keywords:** Molecular biology, Physiology, Diseases, Health care

## Abstract

Mechanical stimulation has benefits for muscle mass and function. Passive stretching is widely performed in clinical rehabilitation medicine. However, the hypertrophic effects of passive repetitive stretching on senescent skeletal muscles against muscle atrophy remain unknown. We used senescence-accelerated model SAM-P8 mice. The gastrocnemius muscle was passively repetitive stretched by manual ankle dorsiflexion for 15 min, 5 days a week for 2 weeks under deep anesthesia. We examined the effects of passive stretching on muscle mass, myofiber cross-sectional area, muscle fiber type composition, satellite cell and myonuclei content, signaling pathways involved in muscle protein synthesis, and myogenic regulatory factors. The gastrocnemius muscle weight and fiber cross-sectional area of the stretched side was found greater compared with that of the unstretched side. Passive repetitive stretching increased the mRNA expression level of Akt, p70S6K, 4E-BP1, Myf5, myogenin, MuRF1.The phosphorylation level of p70S6K significantly increased in the stretched muscles, whereas of Akt and 4E-BP1 remained unchanged, compared to the unstretched side. The Pax7+ cells and myonuclei content did not differ between the stretched and unstretched muscles. These findings suggest that the hypertrophic or suppressed atrophic observation in the stretched muscles are mainly attributable to the protein turnover provoked by stretching. These findings are applicable to clinical muscle strengthening and sarcopenia prevention.

## Introduction

Age-associated loss of muscle mass and function, known as sarcopenia, is characterized by a progressive decline in muscle fiber number and size, a shift in fiber-type composition, and modification of adverse metabolic parameters^1,2^. With the increasing risk of multiple outcomes such as fall-related injuries, physical disability, and frailty, sarcopenia has become a major threat to independence and quality of life in older individuals worldwide^1,2^. Despite the growing recognition of etiologies and clinical importance, sarcopenia is poorly managed by routine treatment. It is imperative to identify effective countermeasures that induce hypertrophy of the senescent muscle and combat sarcopenia progression.

Passive stretching, a type of mechanical stimulus, is widely used in rehabilitation medicine to prevent muscle shortening, maintain the range of joints, and muscle flexibility^3,4^. Some studies indicate that passive stretching may induce muscle hypertrophy by triggering mechanisms such as satellite cells and myogenic growth factors, mechanically activated ion channels, anabolic signaling, and protein synthesis^5–9^. It is performed with minimal risk of injury at relatively light intensity as compared with resistant exercise and is used to prevent muscle disuse or enhance recovery from exposure to prolonged inactivity^8^^,^^10^. However, aging processes alter mechano-transduction, which is the ability for skeletal muscle cells to perceive and respond to mechanical inputs, indicating a perturbed load-induced plasticity for aged muscle hypertrophy^11^. As such, divergences in the stretching protocols applied also affect muscular hypertrophic adaptations. According to previous reports, intermittent stretching performed for 1 week in aged rats resulted in unchanged muscle mass and reduced myofiber size, whereas continuous stretching performed using a splint for 4 weeks provoked an increase in the weight of aged soleus and plantaris muscles^12,13^. Stretching by immobilization induces articular contracture and is not commonly used in routine rehabilitation practice. By contrast, no studies have demonstrated the effect of daily repetitive stretching for short durations on senescent skeletal muscles. Prior reports highlighted the advantages of daily repetitive stretching in regulating the myogenic regulatory factors and suppressing denervation-induced atrophy through the Akt/mTOR signaling cascade^6,7,9^. These findings suggest a potential hypertrophic effect of repetitive stretching, which prevents sarcopenic atrophy in senescent skeletal muscles.

Disrupted protein homeostasis is well established as an initial process that regulates age-related muscle mass reduction^14^. Skeletal muscle atrophy occurs when protein degradation rates transcend rates of protein synthesis^15^. Hence, strategies that enhance protein synthesis may prevent sarcopenic progression. IGF1/Akt/mTOR signaling primarily controls muscle protein synthesis^14,15^. Akt stimulates protein synthesis by activating mTOR and inhibits protein degradation through FoxO-mediated ubiquitin-ligases including Muscle Atrophy F-box (MAFbx)/atrogin-1, and Muscle Ring Finger 1 (MuRF1) downregulation^14,15^. Myostatin, a member of the TGF-β superfamily, has been extensively studied as another potential mediator of sarcopenia owing to its potent negative effects on cell growth and intracellular catabolic and anabolic signaling pathways^16^.

Skeletal muscle hypertrophy can be induced by the activation of muscle satellite cells. Once satellite cells are activated, myogenic regulatory factors (MRFs), including MyoD, Myf5, and myogenin, play a pivotal role in skeletal muscle formation and development^17^. Markers of early myogenesis, MyoD and Myf5, contribute to determination, while the marker of late myogenesis, myogenin, plays a downstream role in muscle cell differentiation to form muscle fibers^17^. However, accumulating evidence suggests that satellite cell numbers and responsiveness are altered during aging, which may be associated with sarcopenia^18^. Although satellite cells are indispensable for muscle regeneration, whether they play a key role in the maintenance of skeletal muscle mass and myofiber size throughout life is still controversial.

Thus, the aim of this study was to investigate the impact of passive repetitive stretching on senescent skeletal muscle mass and fiber morphology by using senescence-accelerated mouse prone-8 (SAM-P8), which exhibits characteristics of accelerated muscle aging, and is reported to be valid for muscular aging research^14^. We also compared changes in terms of muscle fiber type composition, satellite cells and myonuclei content, MRFs, and regulatory factors related to muscle protein synthesis to further elucidate the underlying mechanisms of action. We hypothesized that passive repetitive stretching exerted hypertrophic effects against sarcopenic atrophy in SAM-P8 mice.

## Results

### Muscle mass and muscle fiber cross-sectional area

**Figure 1:**
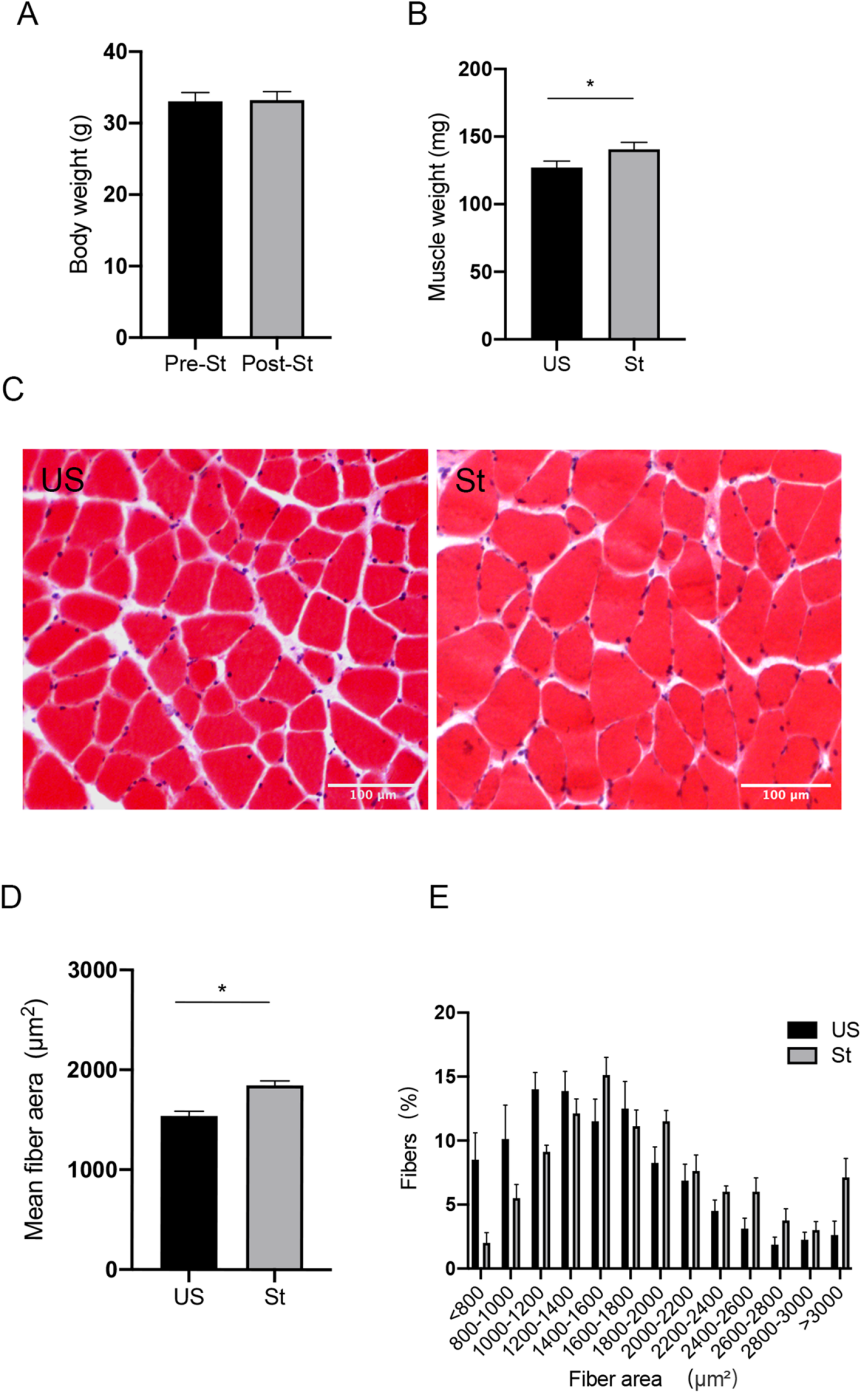
Body weight, muscle weight and fiber analysis (**A**) Body weight. (**B**) Mean weight of gastrocnemius muscle. (**C**) H&E staining of the gastrocnemius muscle. The bar indicates 100 μm. Magnification: ×100. (**D**) Mean cross-sectional fiber area of gastrocnemius muscle. (**E**) Frequency distribution of cross-sectional gastrocnemius muscle fiber area. *US* unstretched. *St* stretched. Values are expressed as mean ± SEM. *P < 0.05, n = 8/group.

There was no significant difference in body weight between the pre- and post-stretching animals (Fig. 1A). The gastrocnemius muscle weight of the stretched side was ~ 10.7% greater as compared with that of the unstretched side (P = 0.028; Fig. 1B). In addition, histological analysis using H&E staining showed that the mean gastrocnemius muscle fiber cross-sectional area of the stretched side was ~ 19.9% larger than that of the unstretched side (P = 0.012; Fig. 1C,D). The frequency distribution of the muscle fiber area further revealed that the stretched gastrocnemius muscle had a shift toward larger myofibers as compared with the unstretched side (Fig. 1E).

### Muscle fiber type, satellite cells and myonuclei

**Figure 2:**
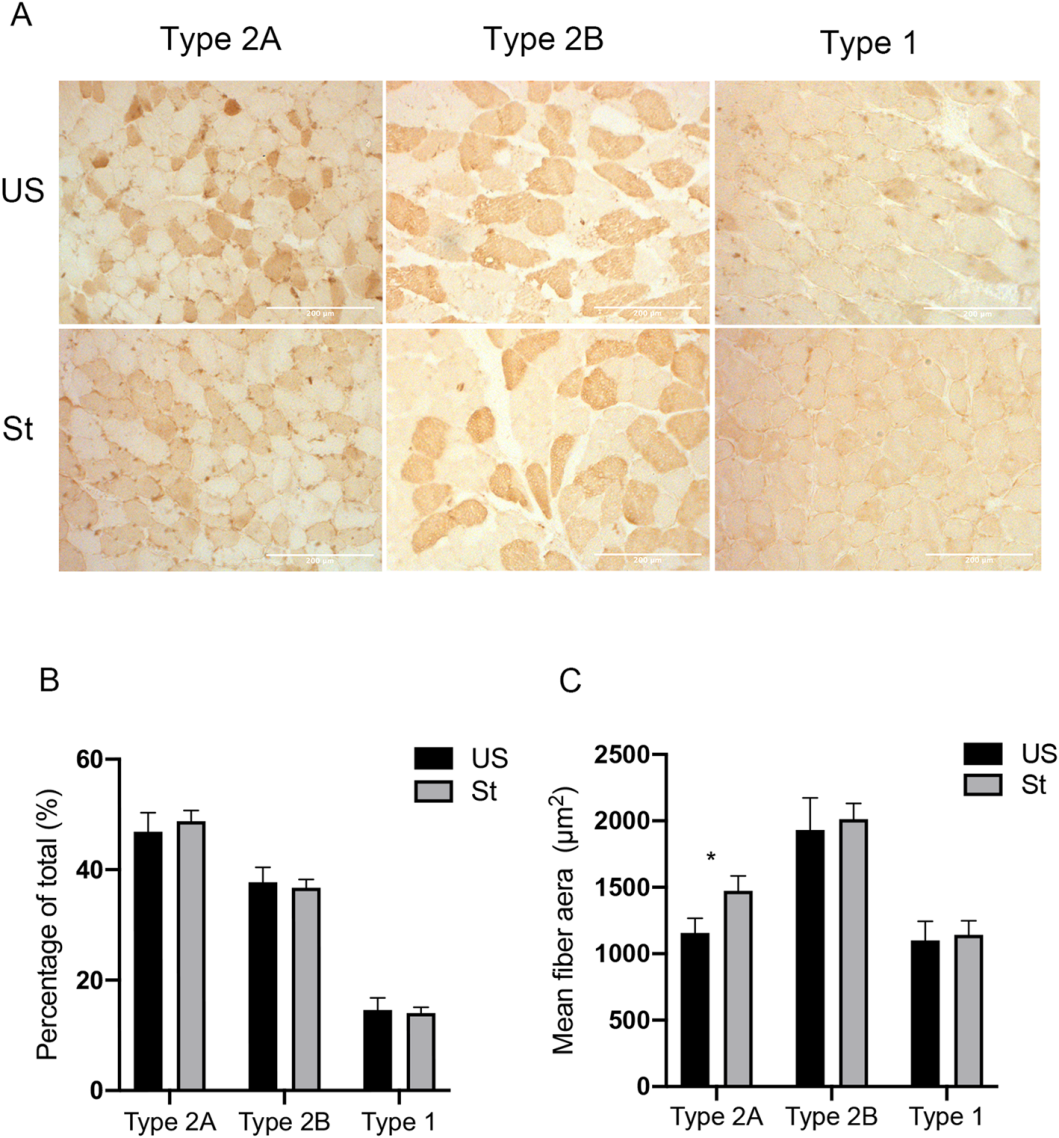
Immunohistochemical analysis of muscle fiber type (**A**) Representative images are shown for type 2A, type 2B, type 1 muscle fiber, visualized with DAB (brown). The bar indicates 200 μm. Magnification: ×100. (**B**) Distribution of fiber types in gastrocnemius muscle. (**C**) Mean cross-sectional fiber area of type 2A, type 2B and type 1. *US* unstretched, *St* stretched. Values are expressed as mean ± SEM. *P < 0.05, n = 8/group.

**Figure 3:**
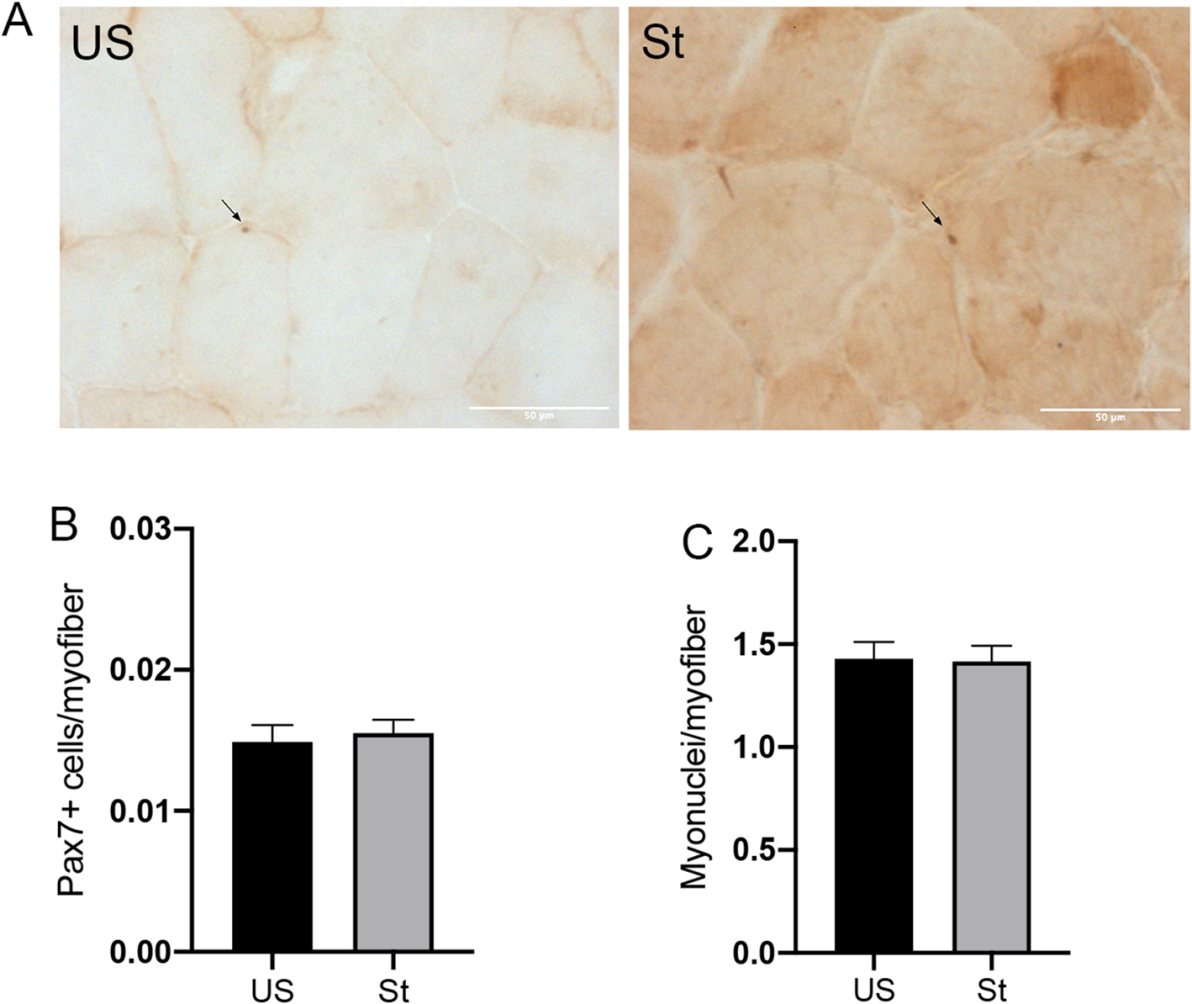
Immunohistochemical analysis of Pax7+ cells and quatification of myonclei (**A**) Representative images showing Pax7+ myonuclei (Arrowheads). The bar indicates 50 μm. Magnification: ×400. (**B**) Quantification of the number of satellite cells (Pax7+ myonuclei/fiber) in gastrocnemius muscle. (**C**) Quantification of the number of myonuclei (myonuclei/myofiber) in gastrocnemius muscle. *US* unstretched, *St* stretched. Values are expressed as mean ± SEM. *P < 0.05, n = 8/group.

No clear differences in the distribution of fiber types were observed when the fiber-type composition was examined with IHC staining (Fig. 2A). Type 2A fibers were the most common in the gastrocnemius muscle, with lower proportions of type 2B and type 1 fibers (Fig. 2B). This suggests that fiber-type switching is not regulated by passive repetitive stretching for a short duration within 2 weeks. In addition, type 2A fibers were mostly responsible for the difference in the cross-sectional area, which was ~ 27.4% greater on the stretched side compared to the unstretched side (P = 0.045; Fig. 2C). Pax7 expression has been recognized as a marker of satellite cells. However, Pax7+ cells were rare, and there was no significant difference in the number of Pax7+ cells between the stretched and unstretched sides (Fig. 3A,B). The number of myonuclei per fiber determined by H&E staining showed no difference between the stretched and unstretched muscles (Fig. 3C).

### mRNA expression of Akt, p70S6K, 4E-BP1, MAFbx and MuRF1, MRFs and myostatin

**Figure 4:**
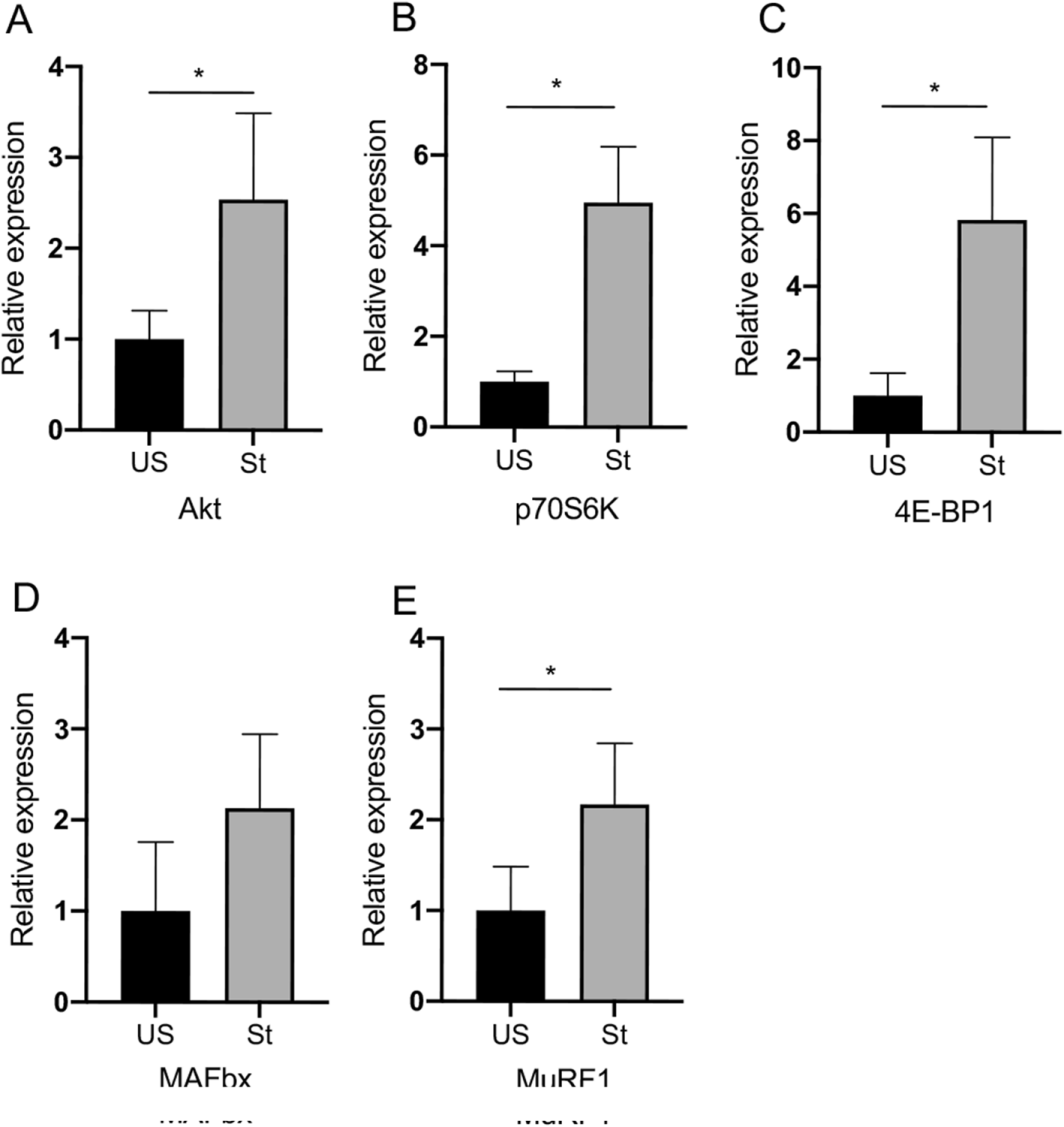
Real-time PCR gene expression of Akt, p70S6K, 4E-BP1, and muscle-specific E3 ubiquitin ligases MAFbx and MuRF1. *US* unstretched, *St* stretched. Values are expressed as mean ± SEM. *P < 0.05, n = 8/group.

As a housekeeping gene, GAPDH was not significantly altered by the intervention. The messenger RNA expression levels of Akt/mTOR pathway signal molecules, Akt, p70S6K, and 4E-BP1 are affected by passive repetitive stretching (Fig. 4). The mRNA expression of Akt increased by 2.5-fold (P = 0.04995; Fig. 4A) on the stretched side as compared with the unstretched side. 4E-BP1 and p70S6K are two downstream targets of mTORC1. The mRNA expression of p70S6K increased by 4.9-fold (P = 0.012; Fig. 4B) and 4E-BP1 increased by 5.7-fold (P = 0.012; Fig. 4C) in the stretched side as compared with the unstretched side. We then measured the expression of major muscle-specific E3 ubiquitin ligases, including MAFbx and MuRF1. The MAFbx mRNA expression was not altered (Fig. 4D), whereas the MuRF1 mRNA expression increased by 2.1-fold (P = 0.04995; Fig. 4E) on the stretched side as compared with the unstretched side.

**Figure 5:**
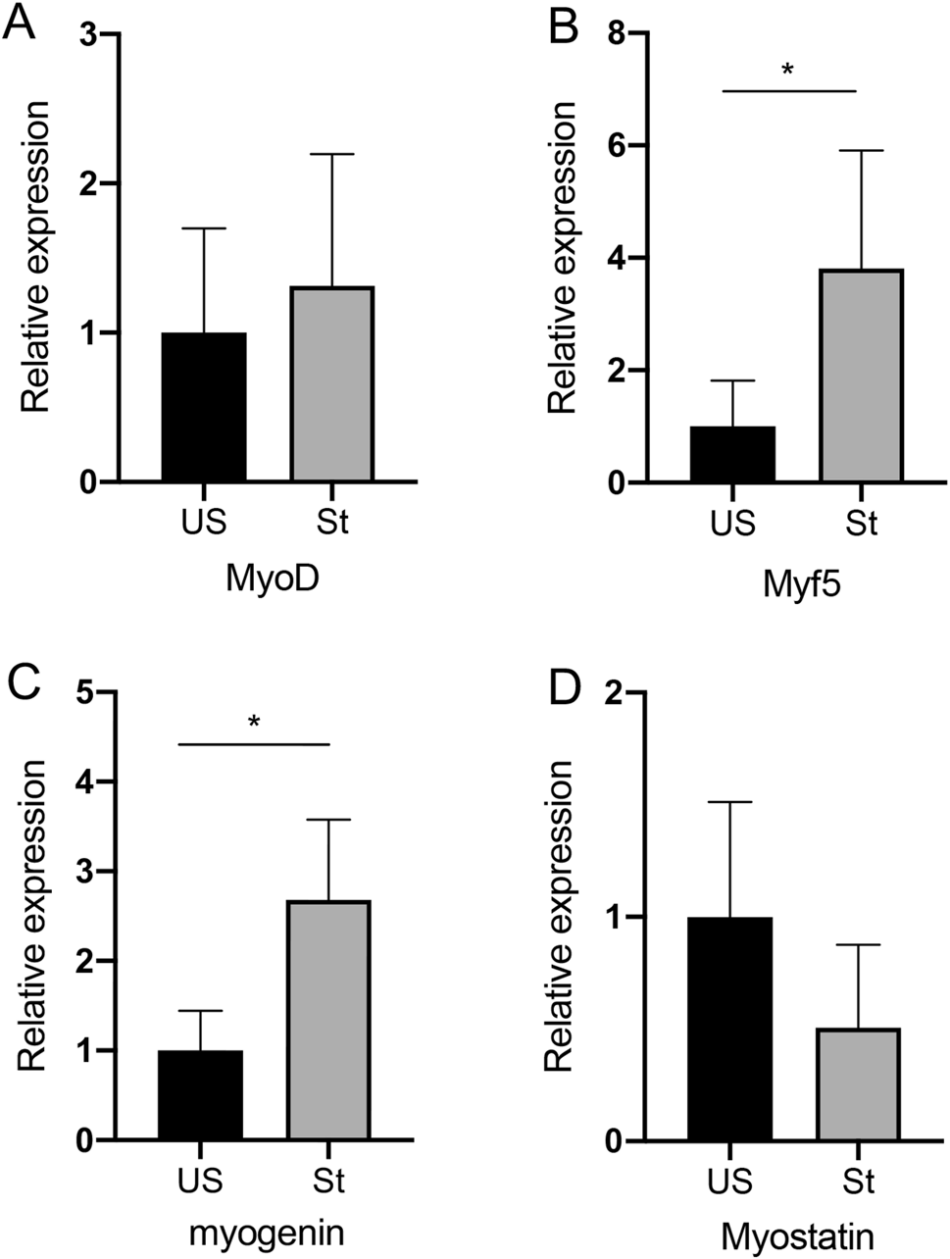
Real-time PCR gene expression of Myogenic regulatory factors and Myostatin. *US* unstretched. *St* stretched. Values are expressed as mean ± SEM. *P < 0.05, n = 8/group.

The expressions of MRFs, including MyoD, Myf5, and myogenin mRNA, are shown in Fig. 5. The MyoD mRNA expression remained unchanged (Fig. 5A). Myf5 increased by 3.8-fold (P = 0.017; Fig. 5B) and myogenin increased by 2.7-fold (P = 0.012; Fig. 5C) on the stretched side as compared with the unstretched side. Myostatin mRNA expression did not differ between the stretched and unstretched sides (Fig. 5D).

### Phosphorylation statuses of Akt, p70S6K, and 4E-BP1

**Figure 6:**
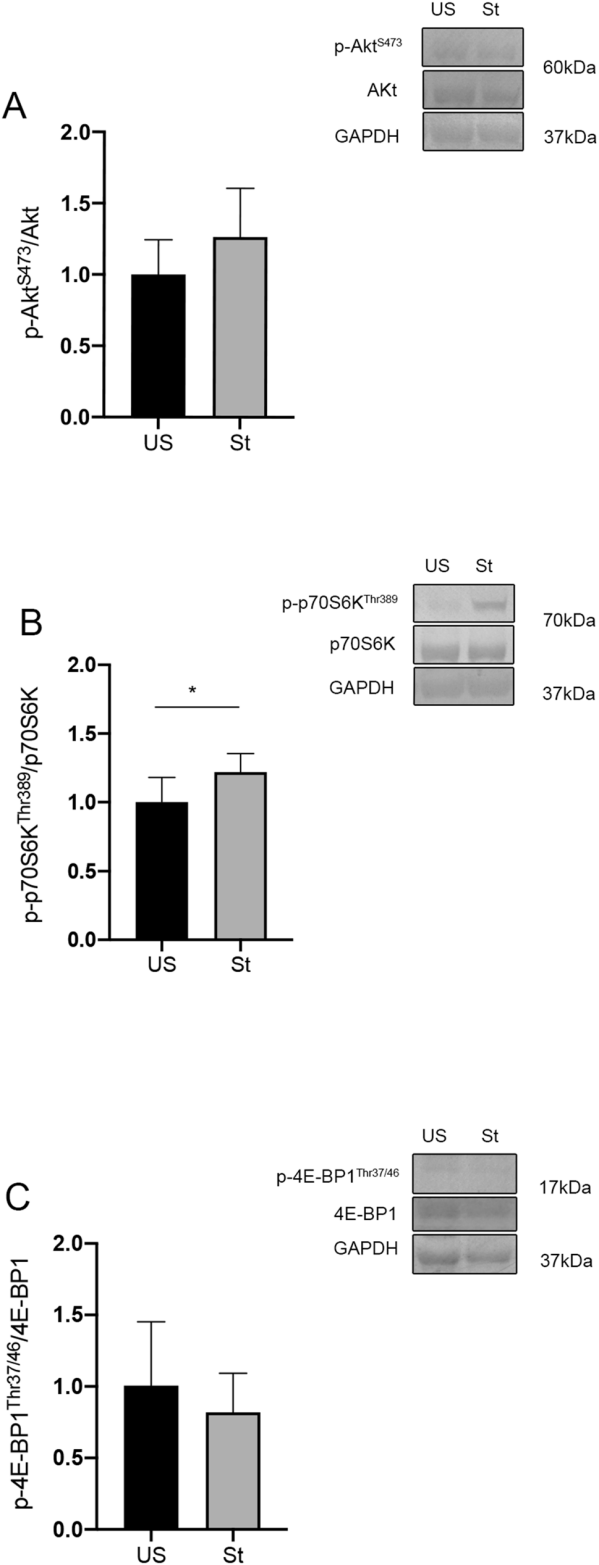
The phosphorylation levels of Akt, p70S6K, and 4E-BP1 measured with Western blotting. Representative western blot images and quantification of phosphorylation levels of (**A**) Akt, (**B**) p70S6K, and (**C**) 4E-BP1. *US* unstretched, *St* stretched. Values are expressed as mean ± SEM. *P < 0.05, n = 7/group.

The level of Akt phosphorylation in the stretched muscles did not differ significantly from the unstretched side (Fig. 6A). The phosphorylation status of the key mTORC1 activity marker, p70S6K, increased significantly by 22% on the stretched side compared to the unstretched side (P = 0.013, Fig. 6B). The phosphorylation level of another mTORC1 downstream target, 4E-BP1, remained unchanged compared to the unstretched muscles (Fig. 6C). Full blot results are in the (Supplementary data).

## Discussion

The findings of this study indicate that passive repetitive stretching for 2 weeks attenuated sarcopenic muscle loss in aged mice and were associated with a greater muscle fiber cross-sectional area, particularly in the type 2A fiber. To the best of our knowledge, this is the first study to demonstrate muscular hypertrophic adaptation to passive repetitive stretching in aged mice in vivo. The theoretical basis for stretch-induced skeletal muscle hypertrophy dates back to in vitro studies. Goldberg et al. found that repetitive stretching applied to cultured skeletal muscle cells provided mechanical stimuli and triggered cellular biomarkers essential for muscle growth^19^. Indeed, robust hypertrophy was observed after progressive stretch overload of the wing muscles in birds^20^. Stretch parameters such as frequency and duration have been identified as important factors that potentially affect the skeletal muscle adaptive process, as the gene expressions involved in muscle growth and atrophy are responsive to the number of stretch sessions^21,22^. Sarcopenia is an age-related reduction in both muscle mass and quality^1,2^. Although prior studies have demonstrated that senescent muscles preserved the capacity to undergo hypertrophy, the ability to perceive and respond to mechanical inputs and translate them into biochemical signals, which is called “mechano-transduction,” was reportedly blunted during aging^11^. The impact of aging on the cellular mechano-transduction process is rooted in multiple factors such as modifications in cell cytoskeleton structures, alterations in mechanosensitive signaling, and the extracellular matrix environment^11^. Zotz et al. found that 1 week of intermittent stretching in aged rats resulted in an unchanged muscle mass, accompanied by reduced fiber size^12^. Hotta et al. reported an increase in soleus and plantaris muscle weights after 4 weeks of continuous stretching without further histological analysis of muscle characteristics^13^. As the rats were awake during continuous muscle stretching, it became difficult to isolate the effects of stretching from isometric contractile activity. In another study, contractions were eliminated by animal anesthesia during stretching, while muscle weight and fiber area remained unaltered^23^. Our results here extend those of previous observations by demonstrating that clinically feasible protocol of passive repetitive stretching is effective in preserving or improving muscle mass and fiber area in the senescent muscles.

Sarcopenia predominantly affects type 2 muscle fibers, whereas type 1 fibers are less affected^2^. In SAM-P8 mice aging from 6 to 10 months, the proportion of fiber type 2A became greater from 53 to 66%, whereas that of muscle fiber type 2B decreased from 42 to 30%, as reported by Guo et al^24^. In addition, the type 2A fiber area increased from 6 to 8 months, followed by a sharp reduction up to 10 months^24^. The remarkable amelioration of the type 2A fiber area with passive repetitive stretching in the present study is notable considering that the preferential atrophy of these high-power generation muscle fibers is a hallmark of sarcopenia progression. As a countermeasure, passive repetitive stretching with a short duration may mitigate or delay the characteristic age-related muscle loss. However, it appears that muscle fiber-type composition seemed not to be susceptible to regulation within 2 weeks of stretching. Fiber-type plasticity within skeletal muscle is regulated by a sophisticated signaling network with two major pathways, calcineurin signaling and AMP-activated protein kinase (AMPK) signaling with a major mediator, PGC-1α^25^. Our observations suggest that the hypertrophic effect of passive repetitive stretching occurred without modifying the fiber-type composition within a short duration of 2 weeks.

The regulation of muscle mass and fiber size substantially reflects changes in protein homeostasis, i.e. the balance between protein synthesis and degradation^15^. Therefore, to decipher the mechanism of action behind the hypertrophic effect of passive repetitive stretching, we first verified whether the Akt/mTOR pathway, which predominantly controls muscle protein synthesis, was involved in muscular adaptation. Akt is an upstream regulator of mTOR, and it is widely recognized that signaling by mTOR is a core module of the pathway through which mechanical stimuli regulate protein synthesis and muscle growth^26^. The regulation is primarily mediated by two downstream targets of the mTOR complex 1 (mTORC1), translational suppressor 4E-BP1 and ribosomal protein p70S6K^27^. Skeletal muscle stretching activates these signaling molecules with elevated phosphorylation levels, including Akt, p70S6K, and 4E-BP1, in vitro^28,29^. Enhanced Akt, p70S6K, and 4E-BP1 phosphorylation were observed in denervated mice soleus muscles when subjected to repetitive stretching in vivo^9^. Several studies have demonstrated that the responsiveness of Akt/mTOR signaling is diminished in overload-induced muscle growth during aging, suggesting limited plasticity for aged muscle hypertrophy^30^. The present study showed that repetitive stretching strongly increased the mRNA expressions of Akt, p70S6K, and 4E-BP1. However, the phosphorylation level of p70S6K was elevated, whereas that of Akt and 4E-BP1 remained unchanged, compared to the unstretched muscles. Previous studies have shown that the phosphorylation status of the key signaling proteins implicated in the regulation of protein synthesis exerted time-course changes in response to a period of acute stretching^8,9^. Thus, a time course study of the acute effect of a single bout of passive repetitive stretching on phosphorylation would be useful to further elucidate protein synthesis regulation induced by stretching of the senescent skeletal muscles.

Moreover, Akt normally blocks the upregulation of several ubiquitin–proteasome genes related to protein degradation in skeletal muscles by negatively regulating FoxO transcription factors^15^. In skeletal muscles, the major muscle-specific ubiquitin ligases include MAFbx/atrogin-1 and MuRF1, which are associated with myonuclear apoptosis and muscle atrophy^15^. However, an expected suppressive effect on MuRF1 and MAFbx was not observed in our study. Peviani et al. found an increase in MAFbx expression in the soleus muscles of rats when daily bouts of stretch were performed^22^. Russo et al. reported that stretching could reduce the accumulation of MAFbx and MuRF1 in a denervated rat skeletal muscle^21^. Furthermore, Soares et al. showed time-course alternations of MAFbx and MuRF1 that decreased drastically after 24-h stretching and then partially recovered after 48- and 96-h stretching in immobilized muscles^31^. These divergent findings suggest that proteasome activity is potentially influenced by stretching protocols or responds differently under physiological and pathological conditions. The alternation of MAFbx and MuRF1 expression in aged skeletal muscle has been reported to be inconsistent. Several studies found that the expression levels of MAFbx and MuRF1 increased in skeletal muscles with aging, which may contribute to sarcopenia^32,33^. However, unaltered and even decreased expression levels of MAFbx and MuRF1 have been shown in other studies^34,35^. In our study, MuRF1 mRNA expression was elevated, indicating the involvement of the cellular degradation pathway in aged skeletal muscle adaptation to passive stretching. Combined with the greater muscle mass and fiber size, this may suggest that hypertrophic or suppressed atrophic observations in the senescent muscles may result from relatively enhanced overall rates of protein synthesis, which possess a superior position in protein homeostasis in the experimental period. Likewise, differential expression patterns of myostatin in stretching have been observed in previous reports^21,22,36^. Myostatin negatively regulates skeletal muscle growth, primarily by acting via activin type II receptors (ActRII), resulting in the activation of Smad signaling^14,15^. Smad signaling suppresses Akt signaling and its downstream effectors such as mTOR and FoxO to regulate muscle growth^14,15^. Alterations in myostatin expression and signaling activity in the context of aging are not completely understood. We could speculate that passive stretching is a potential intervention to counter, at least in part, sarcopenia via myostatin inhibition. However, myostatin expression was not affected by stretching in our study. A further time-course study may help to define the myostatin expression alternation in response to passive repetitive stretching of senescent skeletal muscles.

In addition to protein turnover within individual myofibers, as stated previously, skeletal muscle hypertrophy can also be induced by the activation of muscle satellite cells. In mature muscles, satellite cells are generally quiescent but become activated in response to various stimuli or under muscle regeneration to form new myofibers^37^. When activated, a surge of MRFs, including MyoD, Myf5, and myogenin expression, is required owing to the role of MRFs in driving the differentiation of myoblasts to mature myotubes^17^. Previous studies have shown that mechanical stretching can induce activation of skeletal muscle satellite cells^5^. Elevated expression levels of MRFs have also been observed after short-term passive repetitive stretching^7^. Pax7 expression has been recognized as a marker of satellite cells. To elucidate whether passive repetitive stretching triggered an active regenerative process that may contribute to senescent skeletal muscle hypertrophy, we first sought to detect Pax7+ cells by immunohistochemical analysis. Pax7+ cells were rare, and there was no significant difference in the number of Pax7+ cells between the stretched and unstretched muscles. Therefore, satellite cell content was not stimulated by passive repetitive stretching. As also observed in a human study, satellite cell response during post-exercise recovery is blunted with aging^38^. The expression of MyoD was unchanged, whereas the Myf5 and myogenin mRNA expressions were upregulated in the stretched side when compared with the unstretched side. It has also been reported that MRFs mRNA increases occur in muscles, even in the absence of proliferating satellite cells^39^. Finally, the number of nuclei per fiber was measured in each muscle section to determine whether or not stretching result in satellite cell activation and incorporation of new nuclei. As a result, the stretched and unstretched muscles did not differ in SAM-P8 mice. We are not convinced of the possibility of stretch-induced myogenesis without an increase in MyoD, Pax7 expression levels and new nuclei addition. Overload-induced muscle hypertrophy requires the involvement of satellite cells in growing mice, whereas it is not necessary for hypertrophic growth in mature adult mice^40^.

Our study had some limitations. Considering that SAMP8 mice muscle was not collected before stretching intervention as controls, it remains unclear that how sarcopenic morphological changes progressed during stretching intervention. Therefore, we cannot draw a robust conclusion regarding the extent of the effect of passive repetitive stretching on the muscle mass and fiber area: suppressing the progression of atrophy or inducing hypertrophy. It would also be useful to add time-matched senescence-accelerated mouse resistant-strain 1 (SAMR1) with/without stretching intervention to highlight the difference in muscular adaptation in response to stretching in aged and normal mice. Although pax7+ cells and MRFs related to muscle satellite cells were assessed after the final section of stretching, it is better detected at an early time point in the protocol because of potential time course changes. The time course of the acute effect of a single bout of passive repetitive stretching on the phosphorylation status of the key signaling molecules would be useful to further elucidate protein synthesis regulation induced by stretching of the senescent skeletal muscles.

## Conclusion

Passive repetitive stretching attenuate sarcopenic muscle loss in aged model mouse and are associated with a greater muscle fiber cross-sectional area. Repetitive stretching promotes the gene expression of signal molecules and phosphorylate p70S6K involved in muscle protein synthesis in the senescent skeletal muscles of SAM-P8 mice. Our study suggests that passive repetitive stretching is an effective measure for sarcopenia prevention and maintenance of skeletal muscle mass and function in patients who are exposed to prolonged inactivity, unconscious, or paralyzed (“Supplementary data”).

## Materials and methods

### Animals

We used 35-week-old^24^ male SAM-P8 mice (n = 8; Japan SLC, Hamamatsu, Japan) for this study. They were housed in plastic cages in a temperature-controlled room with a 12-h light–dark cycle and provided free access to water and standard food. The experimental procedures were approved by the Animal Care and Use Committees of Hokkaido University. The study protocol was carried out in accordance with the Fundamental Guidelines for Proper Conduct of Animal Experiments and Related Activities in Academic Research Institutions, under the jurisdiction of the Ministry of Education, Culture, Sports, Science and Technology, Japan. The study was carried out in compliance with the ARRIVE guidelines.

### Stretching protocol

**Figure 7:**
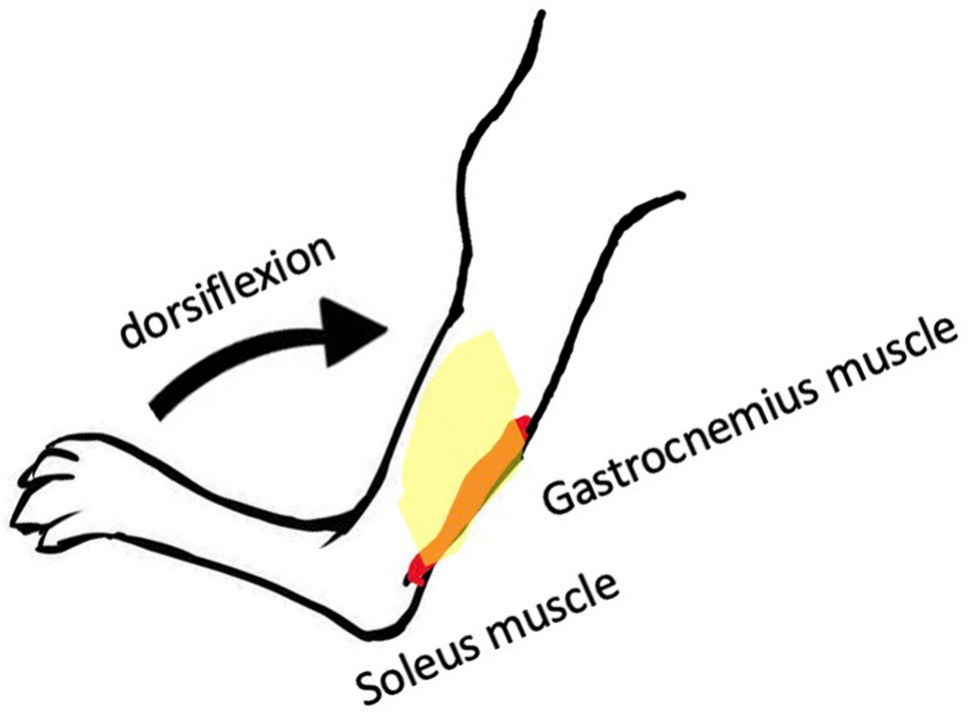
Muscle passive repetitive stretching by ankle dorsiflexion under knee extended position. The images were drawn by Y.W. with Microsoft PowerPoint for Mac version 16.49 (Microsoft, Redmond, WA, USA).

The mice were anesthetized with isoflurane solution (FUJIFILM Wako Pure Chemical Corporation, Osaka, Japan) using inhalation anesthesia equipment (NARCOBIT-E-II KN-1071, Natsume Seisakusho, Tokyo, Japan). The right gastrocnemius muscles were stretched by manual ankle dorsiflexion with knee extended position and within the natural range of motion to avoid muscle damage. Stretching was performed repeatedly, 15 times/min for 15 min daily, 5 days a week for 2 weeks (Fig. 7). Contralateral unstretched muscles were examined as controls.

### Tissue collection and histology

Twenty-four hours after the final stretch session, the gastrocnemius muscles of both legs of the mice were removed under deep anesthesia. After weighing, the gastrocnemius muscles were divided into two blocks. Samples for RNA extraction and western blot analysis were immediately preserved in liquid nitrogen and stored at − 80 °C. Samples intended for histology were collected in Tissue-Tek^®^ O.C.T. compound (Sakura Finetek, Tokyo, Japan) for frozen sections.

SAM-P8 mice gastrocnemius muscle transversal cross-section (8 μm) frozen sections were cut using Cryostat HM550 (Thermo Fisher Scientific, Waltham, MA, USA) and stained with hematoxylin and eosin (H&E) to determine the muscle fiber size and frequency distribution. Tissue slides were observed using PALM MicroBeam IV (ZEISS, Oberkochen, Germany). The cross-sectional area of the myofibers and the number of myonuclei was calculated from 200 myofibers per muscle sample using the ImageJ software (http://imagej.nih.gov/ij/).

### Immunohistochemistry

Muscle fiber type and Pax7-positive nuclei were determined using the ImmunoCruz^®^ rabbit ABC Staining System (sc-2018; Santa Cruz Biotechnology, Dallas, TX, USA) according to the manufacturer’s guidelines. Briefly, 8 μm frozen muscle sections were preincubated for 5 min in H_2_O_2_ and washed twice for 5 min in PBS and blocked for 1 h at room temperature. Subsequently, the sections were incubated overnight at 4 °C with primary antibodies for type 1 muscle fiber (1:500; PAD418Mu02; Cloud-Clone Corp, Katy, TX, USA), type 2A muscle fiber (1:500; PAA755Mu01; Cloud-Clone Corp), type 2B muscle fiber (1:500; PAD416Mu01; Cloud-Clone Corp), and Pax7 (1:100; AP10488B; Abcepta, San Diego, CA, USA). On the next day, sections were washed three times for 5 min in PBS and incubated with biotinylated secondary antibody for 1.5 h. After washing in PBS, the sections were incubated for 30 min with AB enzyme reagent. Sections were then washed and incubated in three drops of peroxidase substrate for 5 min. Finally, sections were washed in deionized H_2_O for 5 min, dipped in 90/95/99.5% ethanol and xylene, and mounted with coverslips using mounting reagent (NEW M·X, Tokyo Garasu Kikai, Tokyo, Japan). Tissue slides were observed using PALM MicroBeam IV (ZEISS). The fiber-type distribution and number of Pax7-positive nuclei per muscle fiber was calculated from 200 fibers using the ImageJ software.

### RNA isolation and real-time polymerase chain reaction

A hand homogenizer with Trizol Reagent (Thermo Fisher Scientific) was used to homogenize the muscle tissues, followed by the addition of 0.2-fold chloroform (FUJIFILM Wako Pure Chemical Corporation), a precipitator to extract total RNA from the supernatant, and the removal of protein and deoxyribonucleic acid.

One-step real-time reverse transcription polymerase chain reaction was performed using the Power SYBR Green RNA-to-CT™ 1-Step Kit (Applied Biosystems, Waltham, MA, USA) with the following steps: 48 °C for 30 min, 95 °C for 10 min, followed by 40 cycles at 95 °C for 15 s, with an annealing and extension step at 60 °C for 1 min. The primers used are shown in Table 1. GAPDH was used as a housekeeping gene.

**Table 1:** Sequences of real-time PCR primers used.

Genes	Forward primer	Reverse primer
MyoD	5′-CCCCGGCGGCAGAATGGCTACG-3′	5′-GGTCTGGGTTCCCTGTTCTGTGT-3′
Myf5	5′-GAGGGAACAGGTGGAGAACTATTA-3′	5′-CGCTGGTCGCTGGAGAG-3′
Myogenin	5′-ACTCCCTTACGTCCATCGTG-3′	5′-CAGGACAGCCCCACTTAAAA-3′
Myostatin	5′-CTGTAACCTTCCCAGGACCA-3′	5′-TCTTTTGGGTGCGATAATCC-3′
Akt	5′-GCCCTCAAGTACTCATTCCAG-3′	5′-ACACAATCTCCGCACCATAG-3′
p70S6K	5′-TGAGTCAAGCCTTGGTCGAG-3′	5′-AAGAGTCGAGAGAGACGCCC-3′
4E-BP1	5′-CGGAAGATAAGCGGGCAG-3′	5′-CAGTGTCTGCCTGGTATGAG-3′
MAFbx	5′-CTCTGCTGTGAGTGCCACAT-3′	5′-CAATGAGCCTGGGTACCACT-3′
MuRF1	5′-TGGAAACGCTATGGAGAACC-3′	5′-AACGACCTCCAGACATGGAC-3′
GAPDH	5′-TGACGTGCCGCCTGGAGAAA-3	5′-AGTGTAGCCCAAGATGCCCTTCAG-3

### Western blot assay

The skeletal muscle tissue (30 mg) was homogenized in 1 mL RIPA lysis buffer (Atto, Tokyo, Japan): 20 mM HEPES, 150 mM NaCl, 1.0% IGEPAL^®^ CA-630, 0.1% SDS, 0.5% sodium deoxycholate/pH 7.5 supplemented with 1 μL protease inhibitor (Atto) and 1 μL phosphatase inhibitor (Atto). The total protein concentration of lysates was determined by incubation for 15 min at 4 °C and centrifugation for 15 min at 14,000*g*. The protein content of the supernatants was quantified with the BCA method (Takara Bio, Shiga, Japan). Bovine serum albumin was used as the standard. Samples were then mixed with dithiothreitol-added EzApply solution (Atto) at a ratio of 1:1 and boiled at 95 °C for 5 min. Subsequently, 7 μl denatured protein (1 μg/μl) per lane was subjected to sodium dodecyl sulfate (SDS)-polyacrylamide gel electrophoresis on 10% polyacrylamide gels (Atto) and then transferred to polyvinylidene difluoride (PVDF) membranes (Atto). After blocking with a blocking buffer (Boster Biological Technology, Pleasanton, CA, USA) for 1.5 h at room temperature, the membrane was incubated with primary antibodies (diluted in TBS-T) against p-p70S6K (Thr 389) (1:1,000; A0533; Assay Biotechnology Company, Sunnyvale, CA, USA) and p70S6K (1:1,000; CSB-PA003686; CUSABIO, Houston, TX, USA), p-4E-BP1 (Thr37/46) (1:1,000; #2855; Cell Signaling technology, Danvers, MA, USA) and 4E-BP1 (1:1000; CSB-PA007994; CUSABIO), p-AKT (S473) (1:1000; CSB-PA000466; CUSABIO), AKT (1:1000; CSB-PA008132; CUSABIO), GAPDH (1:1000; CSB-PA004666; CUSABIO) for 2 h at room temperature. The membrane was washed thrice for 10 min each with TBS-T. Subsequently, membranes were incubated for 1.5 h at room temperature with a diluted secondary antibody (1:1000; Boster Biological Technology) and then washed again in the wash buffer thrice for 5 min each. Protein bands were detected using a DAB chromogenic regent kit (Boster Biological Technology), quantified using Image Quant LAS 4000 (GE Healthcare, Pittsburgh, PA, USA) and ImageJ software, and normalized to GAPDH expression.

### Statistics

The Student's t-test or Wilcoxon signed-rank test were used as appropriate after ascertaining normality of distribution via the Shapiro–Wilk test. A P-value < 0.05 was considered to be statistically significant. Statistical analyses were performed using GraphPad Prism version 8 (GraphPad Software, La Jolla, CA, USA) and SPSS Statistics version 26 (IBM Corp., Armonk, NY, USA). Data are expressed as mean ± standard error of the mean (SEM).

## Supplementary Information

Supplementary Information.

## Data Availability

The datasets generated during and/or analysed during the current study are available from the corresponding author on reasonable request.
