# Supplementary figures and images for: Passive repetitive stretching is associated with greater muscle mass and cross-sectional area in the sarcopenic muscle
Source: Sci Rep. 2021 Jul 27;11:15302. doi: 10.1038/s41598-021-94709-0 (PMC8316451; doi:10.1038/s41598-021-94709-0)

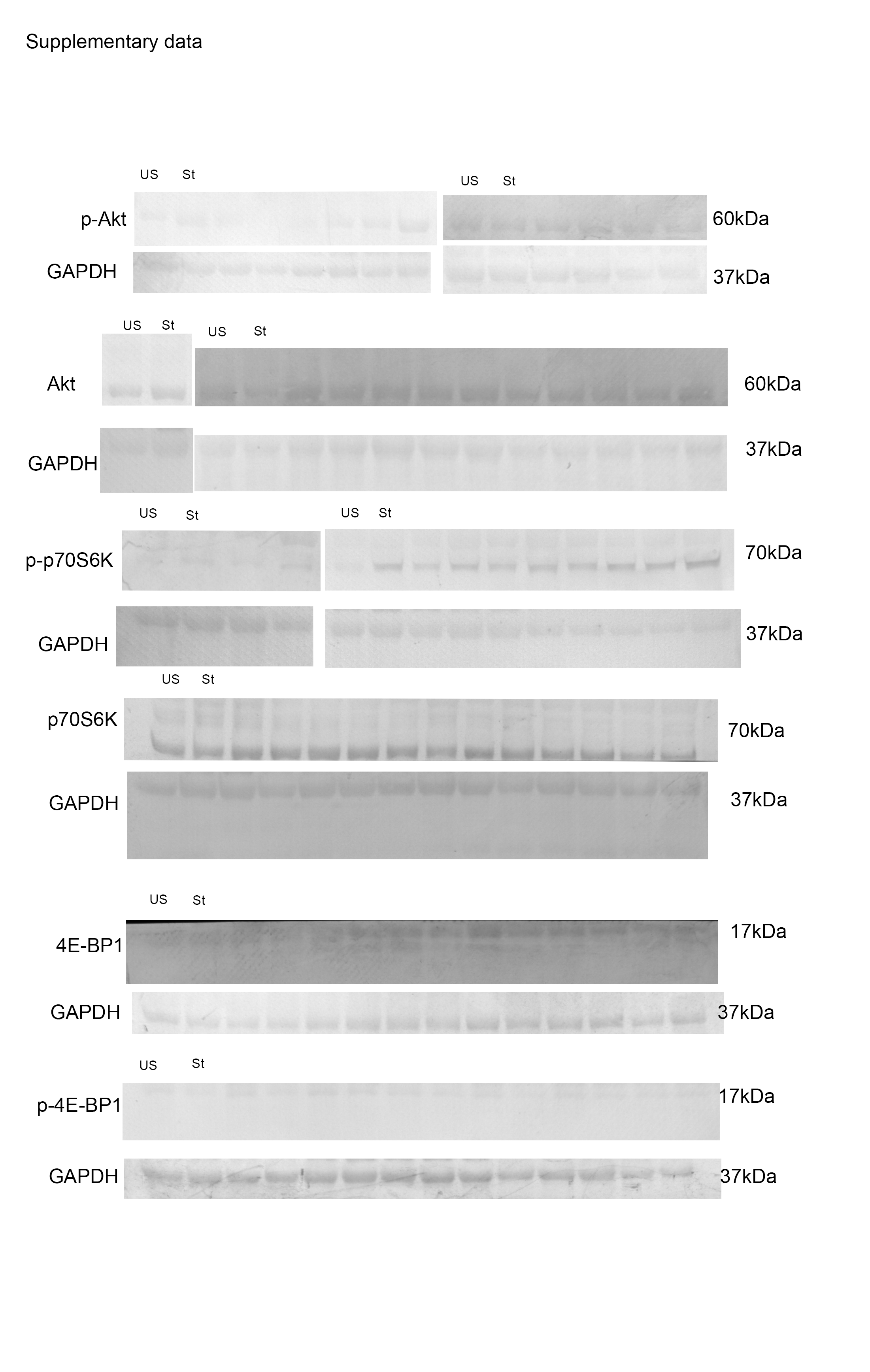

Supplement: Supplementary file 1 — Supplementary Information. [file 41598_2021_94709_MOESM1_ESM.tif]
